# The green beards of language

**DOI:** 10.1002/ece3.506

**Published:** 2013-02-27

**Authors:** Patrik Lindenfors

**Affiliations:** Department of Zoology and, Centre for the Study of Cultural Evolution, Stockholm UniversityStockholm, S-106 91, Sweden

**Keywords:** Chloropogonology, cooperation, Hamilton's rule, language, reciprocity

## Abstract

Language transfers information on at least three levels; (1) what is said, (2) how it is said (what language is used), and, (3) that it is said (that speaker and listener both possess the ability to use language). The use of language is a form of honest cooperation on two of these levels; not necessarily on what is said, which can be deceitful, but always on how it is said and that it is said. This means that the language encoding and decoding systems had to evolve simultaneously, through mutual fitness benefits. Theoretical problems surrounding the evolution of cooperation disappear if a recognition system is present enabling cooperating individuals to identify each other – if they are equipped with “green beards”. Here, I outline how both the biological and cultural aspects of language are bestowed with such recognition systems. The biological capacities required for language signal their presence through speech and understanding. This signaling cannot be invaded by “false green beards” because the traits and the signal of their presence are one and the same. However, the real usefulness of language comes from its potential to convey an infinite number of meanings through the dynamic handling of symbols – through language itself. But any specific language also signals its presence to others through usage and understanding. Thus, languages themselves cannot be invaded by “false green beards” because, again, the trait and the signal of its presence are one and the same. These twin green beards, in both the biological and cultural realms, are unique to language.

## Introduction

Language, according to the *Encyclopedia Britannica*, is “a system of conventional spoken or written symbols by means of which human beings, as members of a social group and participants in its culture, express themselves.” Language is simultaneously information in itself as well as a form of media for storing and transmitting information (Grice 1989; Nowak et al. 2002; Heijdra 2009; Fitch 2010). Information to be stored or transmitted using language needs to be conceptualized within the brain of the transmitting individual, encoded according to rules of semantics and syntax, and then externalized by being articulated into for example spoken or written form (Levelt 1989). In the recipient, the transmitted information undergoes the reverse process, being received, de-coded more or less accurately, and finally encoded into physical representation in the neurons of the recipient (Dennett 1992; Nowak et al. 2001; Fitch 2010). This advanced form of information transfer, requiring a number of specialized biological adaptations, is a feat no other animal can accomplish (Hauser and Bever 2008).

Spoken language is information transmitted over sound waves, requiring levels of de-coding or understanding on top of the interpretation of plain sounds. In its function of enabling information handling, storage and transmittal, language and its associated brain processes can be described as functioning in a very similar manner as an operating system installed on a computer (or a virtual machine: Dennett 1992); translating ‘higher-level’ information into machine (brain) readable code. Researchers are currently investigating the evolutionary processes that have given rise to the syntactic and grammatical rules behind this translation process (reviewed in Fitch 2010).

Cooperating individuals that have the ability to utilize language to exchange information may benefit greatly from this. Through language, cooperating individuals can, for example, coordinate foraging behavior, gang together, alert each other to dangers, gossip, distribute cognition, etc. In fact, the benefits of a communicative system in a social animal are so obvious that they are often taken for granted (Nettle 2006). However, there are also costs associated with language. The ability to speak requires many morphological adaptations, ranging from a specialized descended larynx (but see Fitch 2010) to precise control of tongue, lips and breathing. Language ability also demands increased brain processing power and neurological wiring for accurate and rapid sound perception (Deacon 1997; Hauser and Bever 2008; Heijdra 2009). Further, language opens up for the possibility of false information transfer, of being manipulated by other individuals. This is an evolutionary problem, because if the temptation to cheat is too large, honest communication becomes unattainable (van Baalen and Jansen 2001). Thus, there are clear biological costs of language that need to be weighed against its benefits to fully understand language evolution.

But language does not solely depend on biological adaptations. This is only the biological aspect of language – language ability (the abilities to speak and understand). Language is also a cultural conception where meaning, syntax and grammar develop and are used (Deacon 1997). Any complete theory of language evolution has to be able to account for both the biological and cultural components of language evolution (Nowak et al. 2002; Heijdra 2009). To distinguish between these two aspects, I henceforth use the terms “*language ability*” and “*language itself”* for the biological and the cultural components of language, respectively.

Other authors have made other divisions. For example, Hauser et al. (2002) differentiate between the Faculties of Language in the Broad and Narrow senses (FLB and FLN, respectively), where FLB “includes a sensory-motor system, a conceptual-intentional system, and the computational mechanisms for recursion, providing the capacity to generate an infinite range of expressions from a finite set of elements” and FLN “only includes recursion and is the only uniquely human component of the faculty of language.” For the purposes of exploring the ultimate, evolutionary causes of language evolution, however, distinguishing between the biological and cultural aspects of language are more appropriate, since it allows a clear distinction between the biological and cultural evolutionary processes.

Numerous hypotheses have been formulated for why language evolved (see Számadó and Szathmáry 2006; Fitch 2010 for reviews). These can broadly be divided into three main categories; that language evolved (1) as a by-product, (2) for grooming and bonding purposes, or, (3) to facilitate communication. In the first category it has been argued that language may be a by-product of general thinking abilities (Vygotsky 1986 [1934]; Hauser et al. 2002), that the neural substrate for manual action – through for example stone tool manufacturing – provided a foundation for the evolution of language (Greenfield 1991; Stout and Chaminade 2012), or that language evolved as a by-product of selection for the human ability to sing (Vaneechoutte and Skoyles 1998). It has even been argued that language evolved primarily to structure thinking and was only later co-opted for the purpose of communication (Burling 1993; but see Vygotsky 1986 [1934]), that is that the communicative aspects of language evolved as a by-product. However, as pointed out by Pinker and Bloom (1990) the design of the communication structures in humans is extremely complex, and the only explanation for complex design in nature that we know of is the process of natural selection. Even if language now is irreplaceable as a tool for thinking, its communicative aspects are thus most probably primary (Vygotsky 1986 [1934]; Pinker and Bloom 1990).

In the second category, grooming and bonding, it has been argued that language functions as a form of grooming of group members (Dunbar 1998), for impressing mates (Miller 2000), for bonding with mates (Deacon 1997) or for bonding with offspring (Falk 2004). In the third category, communication, hypotheses focus on the information sharing aspects, that language arose due to the need to coordinate hunting efforts (Washburn and Lancaster 1968; Hewes 1973), that language bonds groups through sharing information about the social life of others (Enquist and Leimar 1993; Power 1998), through the use of gestures (Rowe and Goldin-Meadow 2009), or that language evolved in the context of a so called “asymmetric cooperation,” where information (that was beneficial to the group) was traded for status (Dessalles 1998).

Note that the hypotheses in the last two main categories, grooming & bonding and communication, focus on information sharing; it is only the purpose of this information sharing that differs. In this paper, I will not discuss the issue of exactly what purpose the increased communication proficiency may have served, but will instead assume that one or several such purposes existed. As they are not mutually exclusive it may very well be the case that *all* the purposes listed above are correct. Certainly, language presently serves as an instrument for communication in general, not just for a single specific purpose. There is no real reason to assume that language evolved for communicating something in particular. On the contrary, one characteristic that separates language from other animal communication systems is its general applicability: the ability to express a multitude of meanings. But it is important to keep in mind that the information transferred via language need not be factually correct; language can be used for manipulative purposes as much as for cooperation.

I base my argument below on the observation that language transfers information on at least three levels (1) what is said, (2) how it is said (what language is used), and, (3) that it is said (that speaker and listener both possess the ability to use language). Even if “what is said” may be manipulative, one is always bound to cooperate honestly on “how it is said” and “that it is said.” Language is thus a cooperative endeavor where the encoding and decoding systems have to evolve simultaneously, through mutual fitness benefits.

## Green Beards

Since communication involves at least two participants, language is in its essence cooperation – a cooperative trait requiring at least two individuals utilizing the same medium of communication. Thus, language needs to be evaluated within the framework of costs and benefits of interactions between several individuals, and be evaluated for its fitness effects – both biological and cultural – on both speakers and listeners.

In a biological context, no actor is expected to behave altruistically – wilfully pay a fitness cost to help another – except during specific conditions. This is because a negative effect on the fitness of a genetically coded act means that natural selection quickly should weed out the gene coding for such unselfishness (Trivers 1985). Given that natural selection operates in nature, how then do costly cooperative behaviors evolve? This question vexed Darwin when he formulated his theory of natural selection (Darwin 1859), and it continued to trouble evolutionary biology until 1964 when it to a large degree was solved by Bill Hamilton (1964). Hamilton showed that a “gene for cooperation” can spread in a population only under special circumstances. This is when the fitness cost of helping someone is lower than the probability that the helped individual carries the same “gene for cooperation,” multiplied by the benefit of being helped. This equation has since become known as Hamilton's rule.

Trivers (1971) later built on Hamilton's thinking and applied it also to unrelated individuals who have repeated opportunities for mutual altruism (i.e. cooperation involving temporary costs for the participants). Hamilton's equation has undergone further interpretations in work by for example Nowak and Sigmund (1998), Ohtsuki et al. (2006) and Traulsen and Nowak (2006) (all reviewed in Nowak 2006; Nowak and Highfield 2011), specifying further conditions for when (temporarily) costly cooperation can evolve. Lately, Nowak et al. (2010) have pointed out that spatial considerations may result in the realization that Hamilton's rule only applies under special circumstances and that spatial models may be the key to fully understanding the evolution of cooperation, though it is at present doubtful how much merit there is in this critique of kin selection (see e.g. Abbot 2011; Boomsma et al. 2011; Ferriere and Michod 2011; Gardner et al. 2011; Strassmann et al. 2011).

However, this latter debate may end, one central insight from research on the evolution of cooperation is that if a hypothetical “gene for cooperation” in some way can code for unselfish cooperative behavior that is beneficial to copies of itself in other individuals, it will spread through a population. The conditions under which such a gene will spread are based on probabilities or conditions that the individual being helped also carries a “gene for cooperation.” The assumption underlying these models is thus that the “gene for cooperation” has no way of recognizing itself in other individuals; otherwise no probability or special condition would need to be invoked.

As Hamilton (1964) pointed out in his original articles, the whole problem surrounding the evolution of costly cooperation would disappear if there existed an honest recognition system coupled with the “gene for cooperation” such that individuals carrying the gene could identify other individuals carrying the same gene perfectly. This model was popularized by Dawkins (1976) into what he termed “the green beard effect.” In Dawkins' example, the “gene for cooperation” not only codes for costly cooperation but is also, through for example a pleiotropic effect or linkage disequilibrium, associated with a recognizable trait (e.g. a “green beard” – hence the name). green beards are thus “genes that can identify the presence of copies of themselves in other individuals, and cause their bearer to behave nepotistically toward those individuals” (Gardner and West 2009).

However, selective helping of individuals equipped with green beards is only one of four possible types of green beard effects. The other three are facultative harming (where individuals with green beards selectively harm individuals without), obligate helping (where individuals with green beards help everyone, but only bearers of green beards benefit from this help), and obligate harming (where individuals with green beards harm everyone, but only non-bearers of green beards are actually harmed) (Gardner and West 2009). Both green beards effects proposed below for language are of the “selective helping” kind.

As West and Gardner (2010) have pointed out, “it has been assumed that greenbeards would not occur in nature because they could be easily invaded by “falsebeards” (cheats) that displayed the beard without also performing the behavior” (West & Gardner, p. 1344). Thus, they find only few examples in nature and argue that “Greenbeard genes are likely to be extremely rare in the real world” (West et al. 2011, p. 245). Based on insights from mathematical modeling, however, it has been pointed out that “the green beard effect, in the form of a fluid association of altruistic traits with a recognition tag, can be much more prevalent than hitherto assumed” (Jansen and van Baalen 2006, p. 663). Be this as it may, as we shall see below the twin green beards of language constitute a special case as it is impossible to fake their presence for one simple reason – the cooperative traits and the green beards are one and the same. This fact alone renders false green beards impossible within this system. To make my argument, I will go through the biological and cultural aspects in turn, combining them in the discussion.

### The biological aspect of language – language capacity

The benefits of language in human societies are so great that it is sometimes forgotten that language also incurs costs (Nettle 2006). This may seem counterintuitive as there now exist definite costs of not using language in that it excludes you from a vast communication network and inhibits the potential for distributed cognition (Hutchins 1995). But this is to draw conclusions after the fact. The problem to explain is instead how language evolved initially, when there were no specialized brain structures and no specific morphological adaptations to handle it. It is doubtful whether there currently exists any proto-language in animals (Drobovolsky 1997; Hauser et al. 2002; Heijdra 2009), but whatever the case, the utility of the initial stages of language must be measured in biological fitness costs and benefits, just as for any other biological trait. Language ability already in its initial stages, in its simplest form, is a biological trait requiring morphological speech adaptations and specialized brain components.

To be naturally selected, language ability must have benefited biological fitness in both talker and listener. The obvious candidate for how this came about was that language simplifies and enhances cooperation through information sharing. No matter why you cooperate, the ability to speak and understand will streamline and consequently amplify the results of that cooperation. In one sense, language ability is thus not cooperation in itself, but a trait intimately tied to cooperation that cannot evolve without it. Language was therefore bound to evolve in a social species. In another sense, language is most definitely cooperation, as speaking and understanding can't occur without each other – you have to be cooperating to use language. Herein lies a problem. If there is an opportunity for cheating through language usage, this opportunity will surely be exploited. How then can cooperation be maintained? Crucially, as will be elaborated further below, to be able to communicate anything (the message) you first need to cooperate about the means of communication (the medium). No matter *what* is to be said, you have to cooperate on *how* it is said and *that* it is said. While *what* you say may be deceptive, you cannot be deceptive in *how* you say it or *that* you say it – “cheating” on any of these two levels will only render what you say incomprehensible.

But how do you know who to cooperate with – how do you know who else that possesses language ability? This is where the green beard comes in, because biological language ability already at its crudest would be recognized as language ability by other persons also having this ability. People possessing language ability signal this by using language. This goes for both speaking and understanding; language ability itself is thus a trait akin to a green beard; a costly (through e.g. energy demands on the brain) cooperative trait advertising its own presence. Further, the fact that language ability signals its own presence makes it a green beard impossible to fake. The presences of the biological adaptations for language are recognized on their usage, both when used as the signal (speaking) or as the detection mechanism (understanding). Though the absence of the green beard can be faked (you can pretend to not be able to speak or understand), its presence cannot; the signal of the presence of language ability is impossible to fake. You cannot pretend to speak and understand; language incompetence reveals itself mercilessly.

Importantly, however, language ability is in itself not an honest indicator of trustworthiness when it comes to any other forms of cooperation except what communication system to use. Language may be used for cooperation as well as for manipulation. However, whereas enhanced communication is an obvious advantage in cooperative situations, it is also of utility in certain agonistic interactions. For example, information sharing is the crucial ingredient in the “sequential assessment game” that describes animal fighting (Enquist and Leimar 1987, 1993). This game reveals how animals slowly escalate their conflicts by revealing more and more information about their strength to their opponents. The stepwise escalation minimizes energy expenditure in each conflict, something that is advantageous for both parties. Even conflicts contain elements of cooperation. As phrased by van Baalen and Jansen (2001, p. 218) “if the interaction is detrimental for both partners (as it is, for example, for predators and well-defended prey) then their interests are in line again in that both partners benefit when the interaction is shortened.” Utilizing verbal threats can serve such a function, or even constitute the competition in itself (Miller 2000). Whether agreeing or disagreeing over what is said, disagreeing about the message, parties utilizing language are always cooperating on the means of communication, agreeing on the medium: language.

To summarize the biological side of this discussion, potentially all cooperative interactions, and even some competitive interactions, are enhanced by verbal communication in a social species. Already at the simplest of beginnings, genetically coded language ability signals its existence to other individuals through its utilization. Conversely, speakers will easily recognize language ability in others through their understanding. Even though the information transmitted using language may be manipulative, the ability to speak and understand is not. The signal and the recognition mechanism of the presence of language ability are the same already at the onset, providing a unique potential for biological evolution of language ability. A “green beard” is intrinsic to language ability.

### The cultural aspect of language – language itself

The great utility of human language lies in the possibility of dynamic combination and arrangement of symbols which provides the potential to express an infinite number of meanings (Deacon 1997; Nowak et al. 2000; Nowak et al. 2001; Fitch 2010). The change from static signaling to dynamic information transfer is where language goes from being a biological trait to also being a cultural phenomenon (e.g. Deacon 1997; Nowak and Komarova 2001; Fitch 2010).

However, transferring the insights on the evolution of cooperation from biological to cultural evolution is not completely straightforward. A basic translation of the reasoning in the previous section should result in the question under what conditions a “cultural trait promoting itself” will spread through a population. The condition has to be similar to that formulated by Hamilton (1964); that a “cultural trait that promotes itself” will spread through a population if the benefits to the trait of expressing itself are larger than the costs. This condition will be fulfilled if the trait benefits itself in other individuals. Costs and benefits are here to be understood as decreasing and increasing probability of spreading the trait to other individuals.

In biological evolution, however, a gene can “benefit itself” in other individuals by helping other individuals. In cultural evolution cultural traits can be lost, learnt and re-learnt (Strimling et al. 2009). There is no stage at which a trait is “frozen” in an individual for a fixed amount of time. A cultural trait that makes individuals cooperate with others can therefore be squandered upon individuals who at a later time switch to being selfish. A cultural trait would thus do better if it could distinguish between benefiting the individuals that carries it and benefiting itself directly. This distinction is fulfilled when the cultural cooperative trait and the signal of the presence of that trait are one and the same, that is when the trait is a “green beard,” a condition satisfied for language itself. Language itself favors the cultural fitness (the number of users) of language as a cultural replicator – language itself is an evolving entity in its own right.

Here, it is crucial to point out that the cultural aspect of language has two components. On one hand is the actual information transmitted – the idea encoded in language. On the other hand is the medium through which the information is transferred – the language itself. Think, for example, of a pair of individuals having a heated argument in some language. Though the individuals may not be cooperating with each other, the language they are using is being utilized and is in this way cooperating with itself (Ritt 2004). Language thrives when in use and withers from disuse (Crystal 2000). As an example, the trait “Swedish” is recognized as such by other persons also knowing Swedish. From the point of view of the cultural trait “Swedish,” the preferential treatment – that is the “cooperation” – is simply the use of Swedish. Even if it makes no difference whatsoever for biological fitness whether Swedish or, for example, Swahili is used, the use of Swedish will directly benefit the cultural trait “Swedish” through usage. Thus, using a common language is cooperation even when non-cooperative sentiments are expressed. To have a well-functioning argument there has to be agreement *that* language should be used (language ability) and also an agreement on *what* language should be used (language itself). It is impossible to use language without cooperating on these two levels.

The “medium aspect” of language – the language itself – is recognized on its usage, both when used as the signal (speaking a specific language) or as the detection mechanism (understanding that language). You cannot fake speaking and understanding a specific language – you cannot “cheat” by using Cantonese instead of English in an English-speaking context. If you know a language enough to speak and understand it, you possess that language, and consequently also the green beard. Again, you can cheat about the absence of the ability to speak or understand English, you can fake the *absence* of the green beard, you just cannot cheat about the ability to speak or understand; you cannot fake the *presence* of the green beard. Thus, a “false green beard” is not feasible (but a “false bare chin” is). Using language is what propagates it – an unspoken language will not spread, whereas a commonplace language will spread as the need to know that language for communication purposes increases with the number of speakers. In this way, all languages cooperate with themselves, and identify and favor themselves through usage. Because language itself and the signal of its presence are the same, a “green beard” is intrinsic to language also in the context of cultural evolution.

## Synthesis and Discussion

**Figure 1:**
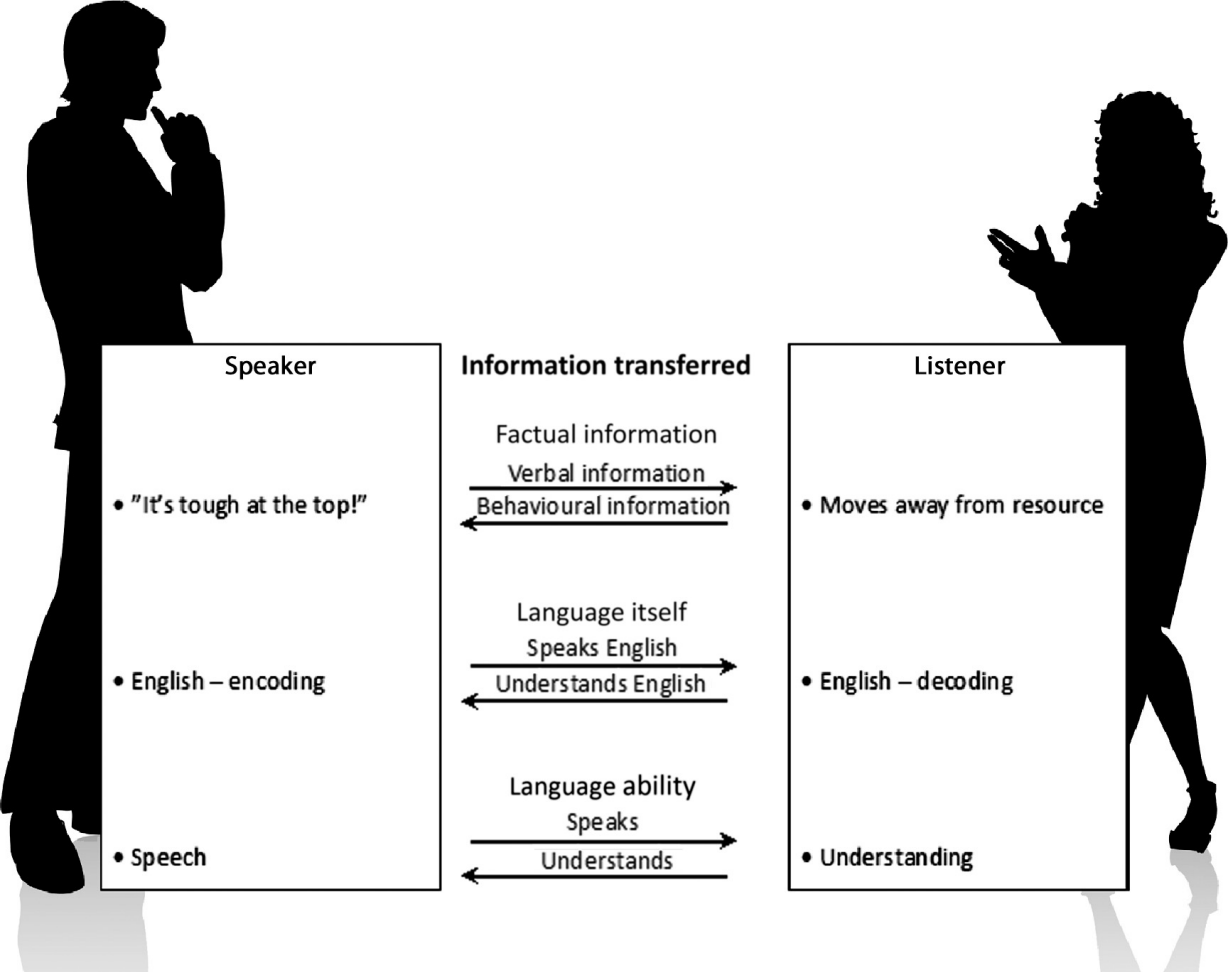
The three levels of information transfer of language. The biological aspect of language – language ability; the ability to speak and understand – reveals its presence through its own expression, through speaking and understanding. These are uncheatable signals, competences that cannot be faked. The cultural aspect of language has two parts. At the “lower” level – language itself; the specific language you are using – presence of knowledge of any specific language is again revealed through speaking and understanding. These are also uncheatable signals, competences that cannot be faked. On this level and the biological level, every act of speech and understanding is cooperation. Lastly, however, there is also the question of the factual information being transferred, which is not an uncheatable signal. With language it becomes possible to manipulate other individuals in a manner simply not possible without language. Crucially, however, to do this requires cooperation on the two “lower” levels.

When utilizing language, information is transferred on three levels, as depicted in Fig. 1. On the most basic, biological level, information of the presence of biological language ability is transferred; the speaker can speak and the listener can understand. But communicating with language also reveals a second, cultural aspect to both parties – what language is being used. The speaker may speak Cantonese, and if the listener understands, she also transfers information that she knows Cantonese. The final level is the information we think of in our everyday use of the term “language” – the information encoded in the language being spoken. Even if there is deception on this level, however, this necessitates cooperation on the two levels below. Language ability and language itself are both cooperative traits that signal their presence through speech and understanding; through usage. Thus, the cooperative traits themselves are the signals of their own presence – green beards. Since the traits and the signals of the traits' presence are the same, false green beards are unfeasible.

The biological aspect of language, language ability, favors its own presence in a population if individuals increase their biological fitness by using language. This will be true in two situations. The first situation is obvious: cooperation on different tasks works better with language than without. Almost regardless of what the cooperation is about, the ability to communicate and share information will aid the possibility of reaching that goal. This is true also in certain conflict situations, as having a common goal is not a necessary prerequisite for having aligned interests (van Baalen and Jansen 2001). But there are of course more problematic cases. In a situation where a speaker manipulates a listener, the speaker's fitness surely benefits, but what about the listener? As van Baalen and Jansen (2001) have modeled the situation, there is a threshold where the temptation to cheat becomes too large, rendering honest communication unmaintainable. But there are two ways to avoid being manipulated by a cunning speaker. First, one can avoid being manipulated through incomprehension. If not understanding what is being communicated, one also cannot be moved to ill-advised action by a well-spoken argument. Were this situation the more common, language would never evolve. The other possibility, however, is to out-smart the speaker by identifying the deception. Interestingly, this would require even better language skills than the deceiver, placing ever higher demands on the cognitive aspect of language ability. Thus, once the threshold to symbolic communication is crossed, selection will act to produce ever more proficient language users (Deacon 1997; Miller 2000).

Crucially, however, the central point of this paper is that even when being cheated in the message part of the interaction, there is necessary cooperation on what medium is being used. Language ability is detectable in both talker and listener to the other party, whether you cooperate, deceive, or detect manipulation. But why call language ability a green beard – a term traditionally reserved for cooperation itself? For the simple reason that whatever way you are interacting, even if you are being manipulated by someone's speech, you are both cooperating on the use of language; information sharing via language is in itself cooperation. Similarly, the cultural aspect of language – language itself – signals its presence through usage and understanding. It is impossible to fake knowledge of (a) language – you cannot use one language in place of another and thus “cheat” – for one party to switch to a language the listener does not understand would only result in communication breakdown. Again, the trait is the signal for the presence of that trait. But language itself can also favor its own presence regardless of if individuals cooperate or compete – language can ‘cooperate with itself’ and through usage sustain and favor its own presence in a population. These two green beards, on both the biological and the cultural aspects of language, make language unique in an evolutionary context.

For a language to be universally expressive, the number of symbol manipulations that are possible to make must reach a certain level, which places new demands on the information processing unit – the brain – to better accommodate and handle the complexity of a dynamic language through ever better cognitive and social learning abilities (Deacon 1997; Fitch 2010). But any such change incurs a cost in that it demands ever more brain power. In humans, around one third of the motor cortex is used to govern movements of the tongue, mouth, face and throat, in comparison to around a tenth in other primates (Gärdenfors 2003). Add to that the areas of the human brain, Broca's and Wernicke's areas, that are specialized for language production and comprehension. Further, advanced general cognitive proficiencies to better handle symbols and better social learning abilities to easier pick up novel language skills and distribute cognition are central abilities for flexible language use (reviewed in Fitch 2010). This sums to rather extensive demands placed on the human brain to handle language competently.

Consequently, the human brain is much larger than that of other primates, as well as extremely metabolically expensive, calculated to require up to 20% of our daily energy intake, in comparison with the more normal 5% of other primates (Raichle and Gusnard 2002). Thus, language is “altruism-like” in that this type of communication is a form of costly cooperation paid for by both parties through higher energy demands. The benefits are usually – but not exclusively (as we saw above) – mutual. The benefits of language, such as the possibility to coordinate behavior, alerting each other to dangers, gossip, or distribute cognition, have to be great indeed to induce such major adaptations. Language has for such reasons been hypothesized to have co-evolved with the human brain and to have been of central importance during human evolution (Deacon 1997).

The transfer of information when using language is, of course, much more complicated than described here, which is a more “bare-bones,” almost autistic, description of information transfer through language use. Add to this description issues such as intonation, gestures, and context and you get a much more complex and complete picture. Though it can be argued that tonal variation is part of language in itself, gestures, body language and context are often considered separately. This complicates things as information is communicated via these means can change the meaning of what is said completely. Similarly, the context of an utterance can change the meaning of that utterance immensely. I have not discussed these complications separately here as these aspects of language may be understood as intrinsic aspects of language that can be added into the “cultural” context discussed above.

Language itself is a cultural trait and is as such only transmitted socially. Once language is present this facilitates further cultural transmission. With language humans are able to transmit information about cultural elements without transmitting the elements themselves: the possibility to instruct someone how to make a hand ax without making the hand ax yourself, the possibility of in 1 h telling someone how to farm without farming yourself for 1 year. Language is in this way a carrier of cultural information, a kind of “genes” of culture. This separation of instruction and element offers some protection for cultural information from copying errors since transmitting instructions instead of transmitting traits is a more reliable mode of transmission (Blackmore 1999). In this manner language functions as a bridge between the biological and cultural evolutionary processes.

Note also that the green beards present in both the biological and cultural sides of the evolution of language make language into a self-referential selective process; a uniquely powerful selective situation akin to Fisherian run-away selection (Fisher 1930; Lande 1981), but even more so since the trait and its detection mechanism are the same rather than separated in two traits in two sexes. The more advanced the speaking becomes; the higher demands are placed on comprehension. Further, the thinking tools facilitated by language can be turned on language itself, as in this paper, giving rise to all sorts of interesting emergent effects (see e.g. Hofstadter 2007).

## Conclusions

Information sharing through language is a form of cooperation. Theoretical problems surrounding the biological evolution of cooperation disappear if a recognition system is present such that cooperating individuals easily can identify each other; that is, if they are equipped with “green beards” (Hamilton 1964; Dawkins 1976). The biological aspect of language – language ability – signals its presence through usage and understanding and is thus bestowed with this type of recognition system. The trait and its signal are one and the same, rendering false green beards are inconceivable.

However, the real usefulness of language comes from its potential to convey an infinite number of meanings through dynamic juggling of symbols (Deacon 1997; Nowak et al. 2000; Nowak et al. 2001; Fitch 2010;). Language itself – as distinct from biological language ability – therefore cannot have evolved as a genetically hard-encoded trait. Thus, language itself is best understood in terms cultural evolution. But language itself also signals its own presence through usage and understanding; it is impossible to fake knowledge of (a) language. Language itself is thus also equipped with a green beard and can therefore benefit its own presence in individual humans regardless of if they cooperate or compete – language can “cooperate with itself”. Again, since the trait and its signal are one and the same, false green beards are inconceivable. Although manipulation and cheating is possible in *what* is said, there is no possibility of cheating in *that* it is said, or *how* it is said – you cannot cheat on language usage.

These coupled signal and recognition aspects of language indicate that the cultural aspects of language co-evolved with language ability, constantly placing demands on ever better cognitive and social learning abilities, simultaneously invoking costs in terms of energetic demands to grow and fuel an ever larger brain (Deacon 1997). The biological evolution of language ability coupled with the cultural evolution of language itself may thus be the best explanation of the historically rapid expansion of the human brain and its abilities, as well as the uniquely human proclivity for social learning.
